# Next-generation Sequencing Extends the Phenotypic Spectrum for *LCA5* Mutations: Novel *LCA5* Mutations in Cone Dystrophy

**DOI:** 10.1038/srep24357

**Published:** 2016-04-12

**Authors:** Xue Chen, Xunlun Sheng, Xiantao Sun, Yuxin Zhang, Chao Jiang, Huiping Li, Sijia Ding, Yani Liu, Wenzhou Liu, Zili Li, Chen Zhao

**Affiliations:** 1 Department of Ophthalmology, The First Affiliated Hospital of Nanjing Medical University and State Key Laboratory of Reproductive Medicine, Nanjing Medical University, Nanjing 210029, China; 2 Ningxia Eye Hospital, People Hospital of Ningxia Hui Autonomous Region, Ningxia 750001, China; 3 State Key Laboratory of Ophthalmology, Zhongshan Ophthalmic Center, Sun Yat Sen University, Guangzhou 510000, China; 4 Department of Ophthalmology, Children’s Hospital of Zhengzhou, Zhengzhou, China

## Abstract

We aim to characterize the clinical features and genetic causes for two affected siblings from a Chinese family with cone dystrophy (CD). Two patients and four unaffected family members were recruited and received complete ophthalmic examinations. Genomic DNA was isolated from the peripheral blood samples from all patients. Targeted next-generation sequencing (NGS) approach followed by intrafamilal cosegregation and *in silico* analyses were employed to determine the genetic defects. Ophthalmic evaluations finalized the clinical diagnosis of CD for the two patients in this family, both of whom presented macular atrophy with no remarkable changes in the peripheral retina. Comprehensive genetic screening approach revealed biallelic missense mutations in the Leber congenital amaurosis 5 (*LCA5*) gene, p.[Ala212Pro];[Tyr441Cys], as disease causative for this family. Both mutations were novel. The first substitution was predicted to eliminate a hydrogen bond and alter the tertiary structure of lebercilin, protein encoded by *LCA5*. We for the first time report novel biallelic *LCA5* mutations in causing CD. Our study extends the phenotypic and genotypic spectrums for *LCA5*-associated retinopathies and better illustrates its genotype-phenotype correlations, which would help with better genetic diagnosis, prognosis, and personalized treatment for CD patients.

Mutations in the Leber congenital amaurosis 5 (*LCA5*) gene (MIM 611408) are reported to cause one to two percent of patients with Leber congenital amaurosis (LCA). LCA is the most severe form of inherited retinal dystrophies (IRDs). Its typical features include blindness or severe visual impairments within the first year of life, congenital nystagmus, sluggish or absent pupillary responses, photophobia, and high hyperopia^1^,^2^. Since its first identification by den Hollander in 2007, *LCA5* mutations have been widely reported as LCA causative in multiple ethnic groups^3^,^4^,^5^,^6^,^7^,^8^,^9^,^10^,^11^,^12^,^13^. Recent studies also indicate the disease causing roles of *LCA5* mutations in two Asian families with early-onset retinal dystrophy (EORD) and a Spanish family with retinitis pigmentosa (RP)^12^,^13^. Patients from the two families with EORD showed dystrophy and pigmentation in the peripheral retina with their fovea spared and central vision preserved, while patients from the RP family showed very poor central vision with remarkable intrafamilal phenotypic diversity.

*LCA5*, mapped to chromosome 6q14.1, contains 9 exons and encodes lebercilin, a widely expressed ciliary protein with high evolutionary conservation^3^,^13^. Despite the ubiquitous expression of lebercilin, lebercilin defects only cause retinal dystrophies, suggesting its important role in keeping regular retinal functions. Herein, according to a comprehensive genetic screening approach, we describe two novel *LCA5* mutations in a previously unreported correlation with cone dystrophy (CD) in two affected siblings from a Chinese family with autosomal recessive inheritance pattern.

## Results

### Clinical Manifestations

**Figure 1:**
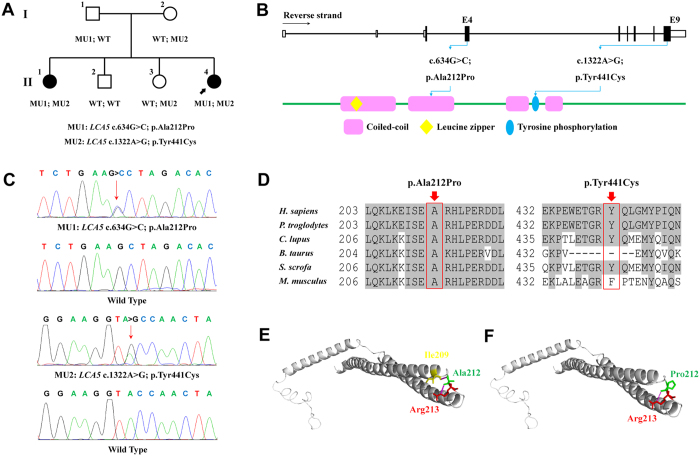
Mutations identified in family YZ. (**A**) Pedigree of family YZ is demonstrated. *LCA5* genotypes are shown for each included individual. Proband is pointed out by arrow. Circles indicate females, and squares, males. Filled symbols indicate affected patients, and empty symbols, normal controls. (**B**) Schematic representation of the relative linear location of the two identified *LCA5* mutations in context of genome structure (upper) and protein structure (below). (**C**) Chromatograms of the wild type and mutant sequences. (**D**) Orthologous protein sequence alignment of *LCA5* from human (*H. sapiens*), chimpanzees (*P. troglodytes*), dogs (*C. lupus*), cattle (*B. taurus*), pigs (*S. scrofa*) and rats (*M. musculus*). Conserved residues are shaded. Mutated residues are boxed and indicated. (**E**,**F**) Predicted crystal structural models of the wild type (**E**) and mutant (p.Ala212Pro, **F**) lebercilin. Residue 212 was indicated in green and its generated hydrogen bonds pink. Residue Ala212 interacts with residues Ile209 and Arg213, while the hydrogen bond between residue 212 and Ile209 was eliminated due to the substitution from alanine to proline.

**Figure 2:**
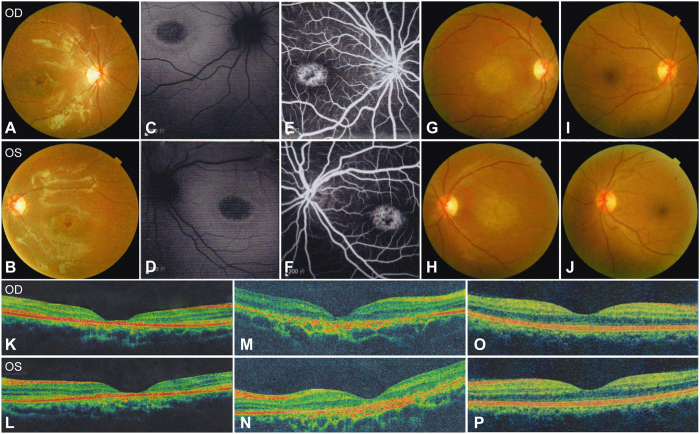
Fundus photographs, fundus autofluorescence (FAF) imaging, fundus fluorescein angiography (FFA), and optical coherence tomography (OCT) findings in the two patients and the asymptomatic mother from family YZ. (**A**,**B**) Fundus of patient YZ-II:4 indicates macular atrophy with loss of fovea reflex but no signs of peripheral involvement. (**C**,**D**) Oval hypofluorescent area in the maculae of both eyes is shown in the FAF of patient YZ-II:4. (**E**,**F**) FFA of patient YZ-II:4 notices speckled changes of increased fluorescence in the macular region of both eyes. (**G**,**H**) Similar to patient YZ-II:4, marked macular atrophy with fovea involved was also found in the fundus of both eyes of patient YZ-II:1. (**I**,**J**) The color fundus of the asymptomatic mother YZ-I:2. (**K**–**P**) OCT presentations of patients YZ-II:4 (**K,L**), YZ-II:1 (**M,N**), and their mother YZ-I:2 (**O,P**). The outer nucleus layer (ONL) and inner/outer segments (IS/OS) layers are vanished in the fovea and its surrounding maculae of patients YZ-II:4 and YZ-II:1 with no remarkable changes detected in the peripheral retina. Retinal pigment epithelium layer was significantly thinned in the maculae of patient YZ-II:1 but was slightly changed in patient YZ-II:4. OD = right eye; OS = left eye.

Two patients, two unaffected siblings and their asymptomatic parents were included in the present study with their family pedigree presented in Fig. 1A and clinical details summarized in Table 1. The 13-year-old proband, YZ-II:4, noticed a decrease in her vision since infancy. Her 30-year-old elder sister YZ-II:1 reported similar onset age and visual symptoms. The other two siblings and their parents denied similar visual issues. No family history of visual problems was recorded. Both patients presented dyschromatopsia since early childhood. The proband also noticed rapid deterioration of central vision. The best corrected visual acuities (BCVAs) dropped from 20/40 at age 6, when she was first diagnosed of CD, to 20/100 within one year, to 20/200 at age 10 and remained stable since then. Similar visual symptoms were reported by her elder sister, who suffered from rapid drop in central vision in her early 10 s and the disease became stable since her mid-10 s. Nyctalopia was not reported and no remarkable changes were revealed in the anterior segment by the slit-lamp examination. Funduscopy indicated retinal atrophy restricted to the macular region with fovea involved for both patients (Fig. 2A,B,G,H). Healthy vascular arcades and optic disk with no signs of peripheral involvement were revealed in the fundus of both patients. Consistent with the fundus photography, fundus autofluorescence (FAF) of the proband indicated hypofluorescent central area (Fig. 2C,D). Fundus fluorescein angiography (FFA) revealed speckled changes of increased fluorescence in the macular region (Fig. 2E,F). Outer nucleus layer (ONL), inner/outer segments (IS/OS), and retinal pigment epithelium (RPE) are significantly thinned as suggested by optical coherence tomography (OCT) presentations of both patients (Fig. 2K–P). Visual field (VF) tests showed diffused loss of central vision in the proband YZ-II:4 and central scotoma in patient YZ-II:1. Photopic responses were undetectable for both patients as revealed by electroretinography (ERG), while scotopic responses were residual. Pathologic responses were recorded by visual-evoked potentials (VEP) tests for both patients, including low amplitude of P100 wave and enlarged latency (implicit time). In summary, the two patients from family YZ presented similar CD phenotypes.

### Genetic Findings

Targeted NGS approach, summarized in Supplementary Table S1, was selectively conducted on patient YZ-II:1. Coverage of the targeted region reached 99.89%, and its mean depth achieved 109.72-fold. A total of 2669 variants, including 2354 SNPs and 315 Indels were initially identified for patient YZ-II:1. Only 6 coding variants retained after filtration against the 6 SNP databases (Supplementary Table S2). Intrafamilial cosegregation analysis further confirmed that biallelic missense variants, *LCA5* c.[634G>C];[1322A>G], were potential disease causing mutations for the two patients (Fig. 1A). Both variants were novel and absent in 150 negative controls. The first substitution, c.634G>C, would lead to the amino acid change from alanine to proline at residue 212 (p.Ala212Pro) of lebercilin. This variation was reveled in the ExAC database with a very low frequency of 0.00001649. Lebercilin contains four coiled coil regions^2^. Residue Ala212, located in the 2^nd^ coiled-coil domain of lebercilin, was highly conserved among all tested species (Fig. 1B,E). Crystal structural modeling of the wild type and mutant lebercilin carrying p.Ala212Pro (residues 154 to 323) were constructed based on the crystal structure of the HP0958 protein (Protein data bank [PDB] ID: 3NA7) with the sequence identity of 27.41% and similarity of 0.33^14^. According to constructed model, the wild type residue Ala212 generated two hydrogen bonds and interacted with two neighboring amino acids, Ile209 and Arg213 (Fig. 1C). The other variation, c.1322A>G, would cause the substitution from tyrosine to cysteine at residue 441 (p.Tyr441Cys), located between the 3^rd^ and 4^th^ coiled-coil regions (Fig. 1B). This variation was revealed in the dbSNP144 database (rs566723830) with the population frequency undescribed. Conservational analysis proved the conservation of residue Tyr441 in multiple species, except for in cattle and rats (Fig. 1E). Taken together, we believe that, rather than polymorphisms, the two identified *LAC5* variants were more likely the disease causing mutations for patients in this family.

## Discussion

*LCA5* mutations have been reported to cause several forms of inherited retinal dystrophies, but have never been found correlated with CD. We, for the first time, report the identification of novel *LCA5* mutations in patients with CD. To the best of our knowledge, this is the first report of the association between *LCA5* mutations and CD, which extends the phenotypic features and genotypic spectrums for *LCA5*-associated retinopathy.

Lebercilin demonstrates a wide expression pattern. In photoreceptors, lebercilin is uniquely located at the cilium which bridges the IS and OS, interacts with intrafalgellar transport (IFT) machinery, and functions as an integral element for the transport of certain proteins through photoreceptor cilia^3^,^13^,^15^. *In vitro* study implies that regular interaction between lebercilin and IFT complex proteins will disappear when human LCA causative lebercilin mutations are introduced, thus disrupting the IFT-dependent protein transport in cilia^15^. In mice models, the inactivation of lebercilin will cause delocalization of opsins and light-induced translocation of arrestin from photoreceptor OS, inhibit the proper formation of photoreceptor OS, and finally lead to photoreceptor degeneration^15^.

Of note, a total of 34 *LCA5* mutations have previously been reported, of which 30 mutations are implicated in the etiology of LCA, 3 in EORD, and one in RP (Supplementary Table S3)^3^,^4^,^5^,^6^,^7^,^8^,^9^,^10^,^11^,^12^,^13^. For patients from the two families diagnosed with EORD, their retinal phenotypes are characterized by widespread RPE atrophy and peripheral intraretinal pigmentation with fovea and central visions relatively preserved, mimicking RP presentations^12^. *LCA5* mutations have not been reported in causing other forms of retinopathies, especially cone dominant dystrophies. Novel biallelic missense *LCA5* mutations, p.[Ala212Pro];[Tyr441Cys], are identified as disease causative for two CD patients in our study, expending the phenotypic spectrums for *LCA5* mutations. Both mutations are absent in 150 unrelated healthy controls. The first substitution from alanine to proline at residue 212 is predicted to eliminate a hydrogen bond and thereafter alter the tertiary structure of lebercilin. A similar heterozygous missense change from alanine to serine at the same residue has been previously found in a Chinese patient with LCA^9^, supporting the clinical heterogeneities for *LCA5* mutations. Noteworthy, most reported *LCA5* mutations that cause severe LCA phenotypes are nonsense, frameshift, or splicesite variations expected to generate truncated lebercilin or cause nonsense medicated mRNA decay (Supplementary Table S3)^16^. Homozygous missense *LCA5* mutation has only been found in a Spanish sporadic LCA patient^12^. Taken together, the cone dominant retinal features in this study are not likely to be correlated with the mutation type or ethnic background. The similar retinal phenotypes exhibited by the two siblings prevent the identification of potential genetic modifiers for lebercilin. Further investigations into intrafamilial and interfamilial phenotypic varieties caused by the same or similar *LCA5* mutations are warranted to assign the genetic modifier for lebercilin, which would help with better illustrations of the genotype-phenotype correlations for *LCA5*-associated retinopathies.

In summary, we have identified novel *LCA5* mutations in the etiology of CD, which extends the phenotypic and genotypic spectrums for *LCA5*-associated retinopathies. The clinical diagnosis for a specific type of IRDs may sometimes be challenged by the phenotypic overlaps among different retinal diseases, or in a young patient with lesser full-bloom of clinical manifestations. It is therefore essential for clinicians to use genetic tests to tell the clinical ambiguity and to prognosticate the disease. Genetic diagnosis also premises gene therapy and other forms of treatments. Our study provides novel insights into the etiology of CD, which would help with the genetic diagnosis, prognosis, and personalized treatment for families with CD. However, future research is still warranted to clarify the pathogenic mechanism underlying how *LCA5* mutations would cause CD and to develop personalized treatments according to each individual’s genetic makeup.

## Methods

### Participants and Clinical Assessments

Four siblings, including two patients and two asymptomatic individuals, and their unaffected parents, were recruited from Ningxia People’s Hospital. Written informed consents were obtained from all participants or their legal guardians. Our study, adhered to tenets of the Declaration of Helsinki, was approved and prospectively reviewed by the Ethics Committee on Human Research at Ningxia People’s Hospital. Each participant received routine ophthalmic examinations, while the two patients and the unaffected mother underwent complete ophthalmic examinations, including assessments of BCVAs, slit-lamp examination, funduscopy, color vision test, VF tests (Humphrey perimetry), VEP, ERG and OCT examinations. FAF imaging and FFA was selectively conducted on the proband, patient YZ-II:4. Additionally, 150 unrelated healthy controls free of retinal or other major ocular diseases were also included. Peripheral venous blood samples (5 mL) were collected from all included participants for genomic DNA isolation with a QIAmp DNA Mini Blood Kit (Qiagen, Hilden, Germany).

### Targeted NGS Approach

Targeted NGS approach was selectively employed on patient YZ-II:1 for mutation identification using a previously described microarray targeting 180 IRDs causative and 9 candidate genes^17^,^18^,^19^,^20^. Library preparation, qualification, NGS on the Illumina HiSeq2000 platform (Illumina, Inc., San Diego, CA, USA), and bioinformatics analysis were performed as detailed previously^21^. Coverage and sequencing depth were further evaluated. Six SNP databases, including dbSNP137 (http://hgdownload.cse.ucsc.edu/goldenPath/hg19/database/snp137.txt.gz.), HapMap Project (ftp://ftp.ncbi.nlm.nih.gov/hapmap), 1000 Genome Project (ftp://ftp.1000genomes.ebi.ac.uk/vol1/ftp), YH database (http://yh.genomics.org.cn/), Exome Variant Server (http://evs.gs.washington.edu/EVS/), and Exome Aggregation Consortium (http://exac.broadinstitute.org/), were subsequently used for the filtration process. Variants found homozygous or with a minor allele frequency (MAF) of over 0.01 in these SNP databases were then discarded. Intrafamilial cosegregation analysis and prevalence test in 150 additional controls were further conducted using Sanger sequencing with the primer information detailed in Supplementary Table S2.

### *In Silico* Analysis

Vector NTI Advance 11 software (Invitrogen, Grand Island, NY) was applied to assess the evolutionary conservation of mutated residue by aligning the orthologous sequences of lebercilin in the following species, including *Homo sapiens* (ENSP00000376686), *Pan troglodytes* (ENSPTRP00000031394), *Canis lupus familiars* (ENSCAFP00000004169), *Bos taurus* (ENSBTAP00000053829), *Sus scrofa* (ENSSSCP00000004819), and *Mus musculus* (ENSMUSP00000034791).

## Additional Information

**How to cite this article**: Chen, X. *et al.* Next-generation Sequencing Extends the Phenotypic Spectrum for *LCA5* Mutations: Novel *LCA5* Mutations in Cone Dystrophy. *Sci. Rep.* **6**, 24357; doi: 10.1038/srep24357 (2016).

## Supplementary Material

Supplementary Information

## Figures and Tables

**Table 1:**
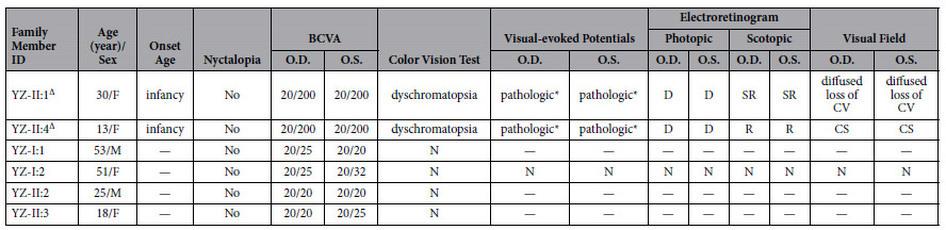
Clinical Features of Attainable Patients.

— = not available; F = female; M = male; BCVA = best corrected visual acuity; O.D. = right eye; O.S. = left eye; N = normal; D = diminished; SR = slightly reduced; R = reduced; CV = central vision; CS = central scotoma.

^∆^Affected patients.

^*^Low amplitude of P100 wave, enlarged latency (implicit time).
