# Supplementary material for: Next-generation Sequencing Extends the Phenotypic Spectrum for LCA5 Mutations: Novel LCA5 Mutations in Cone Dystrophy
Source: Sci Rep. 2016 Apr 12;6:24357. doi: 10.1038/srep24357 (PMC4828721; doi:10.1038/srep24357)
Supplement: Supplementary Information [file srep24357-s1.doc]

**Next-generation Sequencing Extends the Phenotypic Spectrum for *LCA5* Mutations: Novel *LCA5* Mutations in Cone Dystrophy**

Xue Chen, Xunlun Sheng, Yuxin Zhang, Xiantao Sun, Chao Jiang, Huiping Li, Sijia Ding, Yani Liu, Wenzhou Liu, Zili Li, Chen Zhao

*These authors contributed equally to this work.

**Supplementary information file**

Supplementary information file includes three Tables (**Table S1**-**S3**).

| **Table S1 Overview of data production for Patient YZ-II:1.** | |
| --- | --- |
| **Items** | **YZ-II:1** |
| *Target region (bp)* | 1381060 |
| *Raw reads* | 4779322 |
| *Raw data yield (Mb)* | 430 |
| *Reads mapped to genome* | 3435263 |
| *Reads mapped to target region* | 1850232 |
| *Data mapped to target region (Mb)* | 151.53 |
| *Mean depth of target region (X)* | 109.72 |
| *Coverage of target region (%)* | 99.89 |
| *Average read length (bp)* | 89.91 |
| *Rate of nucleotide mismatch (%)* | 0.18 |
| *Fraction of target covered ≥4 X (%)* | 99.80 |
| *Fraction of target covered ≥10 X (%)* | 99.57 |
| *Fraction of target covered ≥20 X (%)* | 99.07 |
| *Capture specificity (%)* | 54.87 |
| *Reads mapped to flanking region* | 155232 |
| *Mean depth of flanking region (X)* | 24.78 |
| *Coverage of flanking region (%)* | 98.56 |
| *Fraction of flanking covered ≥4 X (%)* | 91.22 |
| *Fraction of flanking covered ≥10 X (%)* | 70.88 |
| *Fraction of flanking covered ≥20 X (%)* | 48.22 |
| *Fraction of unique mapped bases on or near target (%)* | 59.27 |
| *Duplication rate (%)* | 25.53 |
| *Mean depth of chrX (X)* | 117.15 |
| *GC rate (%)* | 43.20 |
| *Gender test result* | Female |

| **Table S2 Primer information for confirmation of *LCA5* mutations.** | | | | | |
| --- | --- | --- | --- | --- | --- |
| **Gene** | **Variation** | | **Forward primer (5'-3')** | **Reverse primer (5'-3')** | **Product length** |
|  | **Nucleotide** | **Amino acid** |  |  |  |
| *NPHP4* | c.1930G>T | p.V644L | CACAGGGAGGTGCATCAAT | AGATGGCACTCCCGAATCTA | 387 |
| *LCA5* | c.634G>C | p.A212P | CAAGAGAAAGAACGGGCAAC | ATGCCCAATGAGAAACATCC | 241 |
| *LCA5* | c.1322A>G | p.Y441C | GAATGGGAAAGAGAAGAACTTGA | AGGTAACAATGGCAAAACAGG | 249 |
| *RP1L1* | c.1049C>T | p.T350M | AAGACCAAGCCGAGTGTGAT | AGGGGATTCGTCCAGATTTC | 470 |
| *TOPORS* | c.2987_2988del | p.L996Rfs*10 | GGTGTGCTGGACAAGGAATG | CACAGTCTCTACCAAGACATACTGA | 250 |
| *MFRP* | c.1150C>A | p.H384N | CCCTGTGTCTTCCATCACCT | GTACCCCCAGAGTGTCCTGA | 352 |

| **Table S3 Summary of All Reported Identified *LCA5* Mutations.** | | | | |
| --- | --- | --- | --- | --- |
| **Disease** | **Mutation** | | | **Reference** |
|  | **Nucleotide change** | **Amino acid change** | **Type** |  |
| LCA | g.-19612_-18015del | — | Upstream Deletion | den Hollander (2007) |
| LCA | c.3G>A | p.Met1Ile | Missense | Mackay (2013) |
| LCA | c.42_45del | p.Lys15Glnfs*95 | Deletion | Mackay (2013) |
| EORD | c.69C>G | p.Tyr23fs* | Nonsense | Mackay (2013) |
| LCA | c.103C>T | p.Arg35fs* | Nonsense | Gerber (2007); Corton (2014) |
| LCA | c.142A>T | p.Arg48fs* | Nonsense | Mackay (2013) |
| LCA | c.367C>T | p.Gln123fs* | Nonsense | Mackay (2013) |
| LCA | c.393delA | p.Glu132Lysfs*5 | Deletion | Corton (2014) |
| RP | c.149delA | p.Asn50Ilefs*61 | Deletion | Corton (2014) |
| LCA | c.439_449dup | p.Glu151fs* | Insertion | Mackay (2013) |
| EORD | c.491A>G | p.His164Arg | Missense | Mackay (2013) |
| LCA | c.604T>C | p.Ser202Pro | Missense | Vallespin (2010); Corton (2014) |
| LCA | c.610C>T | p.Gln204fs* | Nonsense | Gerber (2007) |
| LCA | c.633_639del | p.Glu211Aspfs∗13 | Deletion | Mackay (2013) |
| CD | c.634G>C | p.Ala212Pro | Missense | This study. |
| LCA | c.634G>T | p.Ala212Ser | Missense | Li (2011) |
| LCA | c.642delC | p.Leu215Tyrfs*11 | Deletion | Ahmad (2011) |
| LCA | c.720+1G>A | — | Splicesite | Corton (2014) |
| LCA | c.795T>G | p.Tyr265fs* | Nonsense | Mackay (2013); Chen (2013) |
| LCA | c.835C>T | p.Gln279* | Nonsense | den Hollander (2007); Mackay (2013) |
| LCA | c.955G>A | p.Ala319Thr | Missense + Splicesite | Ramprasad (2008); Corton (2014) |
| LCA | c.1062_1068del | p.Y354* | Deletion | Beryozkin (2014) |
| LCA | c.1151delC | p.Pro384Glnfs*17 | Deletion | den Hollander (2007); Mackay (2013); McKibbin (2010) |
| LCA | c. 1186G>T | p.Glu396fs* | Nonsense | Gerber (2007) |
| LCA | c.1207C>T | p.Gln403fs* | Nonsense | Mackay (2013) |
| EORD | c.1231+2insT | — | Splicesite | Mackay (2013) |
| CD | c.1322A>G | p.Tyr441Cys | Missense | This study. |
| LCA | c.1418A>C | p.Glu473Ala | Missense | Corton (2014) |
| LCA | c.1476dupA | p.Pro493Thrfs*1 | Insertion | den Hollander (2007); Walia (2010) |
| LCA | c.1543_1544delinsA | p.Ser515Lysfs*78 | Deletion + Insertion | Mackay (2013) |
| LCA | c.1676C>A | p.Ser559fs* | Nonsense | Mackay (2013) |
| LCA | c.1730dup | p.Leu577Phefs*12 | Insertion | Mackay (2013) |
| LCA | c.1756A>T | p.Lys586fs* | Nonsense | Mackay (2013) |
| LCA | c.1759_1760insAG | p.Leu587Glnfs*7 | Insertion | Mackay (2013) |
| LCA | 1820_1821del | p.Gln607Valfs*6 | Deletion | Li (2011) |
| LCA | c.2011C>T | p.Arg671fs* | Nonsense | Mackay (2013) |
| LCA = Leber congenital amaurosis; EORD = early onset retinal dystrophy; RP = retinitis pigmentosa; CD = cone dystrophy. | | | | |
